# Mapping the production-consumption gap of an urban food system: an empirical case study of food security and resilience

**DOI:** 10.1007/s12571-021-01142-2

**Published:** 2021-02-08

**Authors:** Paul D. Jensen, Caroline Orfila

**Affiliations:** School of Food Science and Nutrition, University of Leeds, Woodhouse Lane, Leeds, LS2 9JT UK

**Keywords:** Cities, Foods system, food security, Self-reliant, Resilience, Circular economy

## Abstract

Urban food systems are complex and increasingly recognised as not being sustainable, equitable or resilient. Though globalisation and lengthening of agrifood supply chains has brought many benefits, such as year-long availability of fresh produce and modernisation opportunities for some developing regions, they have increased reliance on food imports and reduced the food and nutrition resilience of many cities. This premise has been widely witnessed following recent financial, climatic and pandemic driven disruptions to food supplies. A greater understanding is thus needed of the lived reality of a modern city’s ability to sustainably and equitably feed itself in a crisis situation or otherwise. In a changing world, such knowledge is valuable on a variety of strategic planning levels. Employing publically available data, the scale of food security and resilience, and options for their improvement, are holistically assessed through a case study spatial analysis of the urban food system of the city of Leeds in the United Kingdom. The case study found that the Leeds city region is home to a significant and diverse food production and provision system, but it is not food secure in terms of providing sufficient energy or macronutrients, or functioning in an equitable manner for all of its residents. Options for improving the performance of the system, including urban farming and industrial symbiosis, were found to be nuanced and would only be effective alongside a range of complimentary interventions as well as high levels of investment, multi-sector cooperation and strong governance. Though food system evolution and development are grounded in local context, the methods, general findings and circular economy focussed recommendations emanating from the case study, are widely applicable.

**Supplementary Information:**

The online version contains supplementary material available at 10.1007/s12571-021-01142-2.

## Introduction

### Cities and food systems

Modern cities are large complex systems. Their form and function are dynamic products of human interactions with the local and wider technical, geophysical and biological environments. They are increasingly becoming home to people across the globe, with more than 55% of the global population living in urban areas (UN 2018). Though their growth is slowing compared to developing regions in Africa and Asia, rapid urbanisation over the last two centuries has pushed this figure to 74% and 82% in Europe and North America respectively (eurostat 2018). From a variety of social, economic and technical perspectives, these growing cities are open systems that rely on a continual supply of resources to function and provide their increasing number of residents with services (Bretagnolle et al. 2006; Rees 2019). In a period of global uncertainty, underlined by hastening climate change and recent global financial and pandemic driven shocks, the sustainability and resilience of these systems is however increasingly questioned.^1^ Such questioning is particularly true of the food systems that the residents of cities and their urban conurbations all intrinsically depend on (Grewal and Grewal 2012).

Food systems can be seen as a local sub-system of the city, as a global system in their own right or indeed as an interacting multilayered combination of both (FAO 2018; Zhang et al. 2018). As a concept and entity, food systems go beyond aspects of production, provision and consumption, and encompass the wider social, economic and environmental impacts and outcomes of these activities (Kennedy et al. 2018). Moreover, the concept of the food system recognises the interrelatedness of system components, the agency they exert on each other and myriad emergent properties that impact on the local and global environments. Over recent years, this complexity and interconnectedness of the global food system, and many countries dependence on global imports, has increased (Kumma et al. 2020). Indeed, at the city scale, it has been suggested that to meet basic daily needs, modern cities “*almost exclusively*” rely on imports of resources (Grewal and Grewal 2012: 1). The reliance on imports, national or global, and by definition not being self-reliant, is an undesirable characteristic from a resilience and risk management perspective and leaves the system open to damaging shocks. Food ‘deserts’ and a lack of urban self-reliance in food production is, however, common and has been widely acknowledged particularly within areas of the United Kingdom, mainland Europe and the United States, where urbanisation dominates but food provision does not typically shape wider urban planning (e.g., Morgan 2009). With long standing concerns that the populations of Asia and Africa are growing quicker than the food systems can adapt, concerns about urban development and food planning and governance, or lack thereof, could be applied globally.

The drivers and outcomes of actual or potential reliance on imports are multifaceted rather than an intentionally developed system property (Nayak and Waterson 2019). Aside from a simple outgrowing of the food production and provision capacity of settlements that were established decades, centuries and even millennia ago, largely located to exploit local natural assets such as fertile soils, mineral deposits, clean running water and/or easy access to the opportunities provided by the sea, the consolidation of food outlets into supermarkets, advances in logistics and consequent globalisation have played a large role in this reliance on imports. Indeed, in cities the loss of small local food outlets, and a greater reliance on global supply chains, has coincided with the establishment of supermarkets and their provision of goods via cost-effective and competitive supply chains (Jennings et al. 2015). Though there are positives to these global food supply chains, such as the supply to colder European countries of year-round nutritious fruit, seeds and nuts that only grow in appreciable quantities in warmer climates, and the creation of jobs and business opportunities in developing regions, they can also increase the carbon footprint of food logistics and transfer other significant direct and indirect environmental and social impacts to other regions (Carter and Roelen 2017; Kennedy et al. 2018). Discussing the multitude of environmental and socioeconomic impacts of global supply chains is beyond the scope of this article. However, from a basic product ethics, security and safety perspective, it is not advisable to be overly reliant on long and sometimes opaque supply chains for contentiously produced goods or products produced within areas subject to volatile climatic and/or political environments (Grimm et al. 2014, 2016; Jensen et al. 2016; Cottrell et al. 2019; Garnet et al. 2020). This is particularly true in a modern era of ‘just-in-time’ food supply chains that are employed for reasons of process efficiency and the minimisation of perished produce, but rely on a complex supply-demand coordination and communication effort that is vulnerable to any friction along the chain or shocks to the wider system^2^ (Garnet et al. 2020).

Aside from the ethical and sustainability questions that arise from increasingly transferring the negative impacts of industrial food production and provision away from cities to other areas, sometimes thousands of kilometres from the source of demand, the above narrative raises the question of how resilient cities are in respect of being able to feed themselves in an equitable and long-term sustainable and resilient manner. Though the question of whether a city region can feed itself is in some respects a theoretical one, climate change and sudden system shocks emanating from climatic events such as extreme rainfall, natural disasters such as earthquakes, and pandemics such as the one caused by COVID-19, that have led to documented cases of food insecurity and losses of nutritious produce (e.g., milk poured down drains, crops rotting in field and empty supermarket shelves from disruptions to labour and logistics (Cagle 2020; Harvey 2020)), have directly shown how fragile food provision can be.^3^ As such, theoretical or otherwise, a greater understanding of how food resilient a given city potentially is (or can be), is important from strategic local resource security and public health perspectives. Indeed, from a public health and food system perspective, it should be remembered that food does not equate to nutrition (e.g., MacDiarmid et al. 2018). As such, an understanding of a given city region’s *nutrition* demand and local supply resilience is in some respects as equally important as the ability to provide a city’s residents with sufficient daily calories - in a crisis situation or otherwise. Given the complexity of food systems, their numerous interacting components and interrelated outcomes, assessing levels of equitable, sustainable and resilient local food self-reliance is, however, not an easy task.

### Exploring urban food systems

The exploration of food systems and their performance requires a holistic approach that acknowledges the various elements of the system and their interactions and, perhaps more importantly, the respective drivers of their outcomes and impacts. For instance, beyond climatic forces and physical resource limitations, it must be recognised that supply and demand within a system is not static or homogenous, nor is supply and demand driven by one set of values or needs. From an agency perspective, the system is home to several overarching actors. 1: those who produce food; 2: those who process food; 3: those who procure food, and 4: those who consume food, all of which have different demands on the system that are shaped by their respective fundamental needs and values. To complicate matters further, there are those who fill numerous roles and transcend positions in the system; for instance, everybody consumes food but some also produce, process and/or engage in procurement of locally available food, whether it is produced in the city or otherwise. Work on such nuance of supply and demand agency within food systems is not new. In particular, the Food and Agriculture Organization (FAO) and their partners, the Resource Centre for Urban Agriculture and Forestry (RUAF), have undertaken extensive work on City Region Food Systems that emphasise the need to undertake assessments that are rooted in local context and policy (e.g., RUAF 2015).

The studies of the FAO/RUAF have been undertaken on several continents and in both developed and developing regions (e.g., in Colombo, Sri Lanka, Asia; Dakar, Senegal, Africa; Toronto, Canada, North America; and in Utrecht, The Netherlands, Europe) (FAO 2020). The goal of these food system studies was to understand ways of improving the sustainability of food provision across cities and their urban fringe while increasing links to rural areas and providing more people with access to healthy and affordable food and social development opportunities (e.g., new skills, new jobs and business opportunities). Overall, despite acknowledgement that each system is rooted in local context, some common challenges were identified across the city-region studies (Dubbeling and Santini 2018). The first common challenge, across nations, encompassed issues around the existence or availability of empirical data of the quality required to understand the performance of the system and, hence, its outcomes and impacts. The second common challenge was the difficulties of maintaining engagement with the myriad of stakeholders involved in a city-region food system due, for example, to participation fatigue, competing interests and/or failure to suitably embed the relevance of system improvements at the outset of activities. The last shared challenge was seen to be governance; largely in respect of the complexity of governance levels and the respective planning policy and priorities at each level. Though not universally witnessed, other shared themes and experiences were identified within case study cities; particularly in respect of the provision of locally produced food in cities that perhaps outwardly would or could be expected to be well catered by local production efforts.

The two RUAF studies within Toronto and Utrecht arguably best highlight the contradiction of regions that possess significant and productive food systems but are largely reliant on imports. Though Utrecht may be well provisioned in absolute food terms and surrounded by significant production and processing activities, the city was found to lack access to an appreciable level of locally produced food with 90–95% of Utrecht‘s food coming from outside of the ~500km^2^ of the Utecht-10 region (i.e., Utrecht and 9 surrounding municipalities) (Haenen et al. 2018). Perhaps more notably, concerns were raised over the diminishing quality of local diets and that locally produced food that was available within Utrecht was largely purchased by higher earners and/or those possessing a higher education (i.e., not necessarily accessible to the less fortunate in society). Though much of the city-region’s reliance on imports is a product of Utrecht’s vast urbanisation, it was noted that local agricultural produce largely consisted of dairy products, with local vegetable production being “*almost non-existent*” (Haenen et al. 2018: 9). Despite being home to 20,000 farms and possessing a greater diversity of local production, the Toronto city-region food system was similarly found to rely on food that, on average, was sourced from close to 4500 km from the city (Miller and Blay-Palmer 2018). Despite its own local demands driven by urban growth, much like the Netherlands (that is deemed a leader in food innovation and is more self-reliant than many nations, e.g., Viviano 2017), much of what is produced in the greater Toronto area is exported with, paradoxically, similar produce being imported (Miller and Blay-Palmer 2018). The Toronto RUAF study also highlighted, similarly to Utrecht, concerns with communities having equitable access to healthy locally produced food, with reports of hunger and up to 17.6% of residents being food insecure alongside ~3.5million residents self-reporting as obese (2018: 26).

Dealing with deficiencies in local food supplies, or simply making them more sustainable and equitable was not seen as easy within either region. For Toronto it was surmised that there was a need for more mid-scale processing and distribution infrastructure, such as local food hubs, that are supported by scale appropriate regulations and, where appropriate, financial support mechanisms that ensure everyone can eat locally produced healthy food (Miller and Blay-Palmer 2018). In general, however, a review of each of the FAO/RUAF case studies suggests system development activities within all regions can be said to have generally revolved around three key areas: expansion of the system to create a more productive relationship with their rural surroundings; optimisation of what is already happening and in the city-region system, including reduction and better use of system waste; and, finally, increasing production within the city. Notably, in recent years the latter option has been a particular subject of interest for a variety of researchers. Beyond the work of third-sector organisations, studies on food systems and particularly the food security and resilience of cities, and how this can be improved, has been considered within academic research. For example, Grewal and Grewal (2012) considered the ability of the city of Cleveland, Ohio, to feed itself in a healthy and sustainable manner and at options for boosting production, finding that the city was currently only 0.1% self-reliant (in terms of total spend) on all food and beverages. Though this figure was solely derived from inner-city community gardens rather than the wider food system, any improvement in local provision would improve the city’s health and environmental and economic sustainability. By exploiting vacant land within the city it was suggested that urban farming using conventional methods could boost self-reliance to between 4.2% and 11.1% - employing some innovation in the form of hydroponics it was found that these figures could be further increased to between 7.4% - 17.7% with the potential for 22% and 100% self-reliance in fruit and vegetables using conventional and hydroponic systems respectively (see Grewal and Grewal 2012: 9). It was acknowledged, however, that achieving such hypothetical localisation of food production would, aside from significant finance, require a variety of resources (e.g., land, nutrients, buildings, water) that, within a city, may have numerous competing demands. Ackermann and colleagues (Ackerman et al. 2014) came to similar conclusions for New York, highlighting that the city faces a food related health crisis which could, notwithstanding numerous challenges, potentially be alleviated by urban agriculture and healthy food production, noting that the areas of the city best suited to such production activities were also those suffering the greatest food insecurity and inequality.

Further highlighting the international nature of the problem being addressed, discussions over the self-reliance, resilience and equitability of food systems have also been addressed within the UK, with urban agriculture again being touted as a route to increasing the system’s productivity and improving the social aspects of urban food production. Grafius et al. (2020), in Bedford, Luton and Milton Keynes, and Edmondson et al. (2020a, 2020b), within the large cities of Leicester and Sheffield, each acknowledged issues over food security within their respective study areas, highlighting through use of a Geographic Information System options for increasing production within existing greenspace, particularly allotments and gardens. Though not providing a measure of each of the cities food demand-production gap, each paper highlighted wider socioeconomic and environmental benefits of increasing resilience and closing the ‘gap’ with a particular focus on increasing availability of fruit and vegetables in areas of such need. Indeed, for Sheffield, an area possessing significant levels of deprivation, the study identified an additional 98m^2^ of land per person that could potentially be employed in urban horticulture, albeit acknowledging that 71m^2^ of this area was comprised of gardens of which much would not be available (Edmondson et al. 2020b). The authors noted, however, that repurposing just a quarter of the identified city’s greenspace would go a long way toward providing the cities daily fruit and vegetable needs.

The food system work undertaken in Utrecht and Toronto by RUAF, and to some extent by researchers within the United States and the UK, highlight attempts to understand the performance of the food systems of modern cities. They identify both problems and solution to raised issues, whether that is in the form of increased coordination of existing local producer activity with the intention of getting locally produced food on local plates, or through vastly increased urban production to improve self-reliance. Possibly due to the stated challenge of data availability, most food system studies, however, provide little in the way of an empirical assessment of just how big the production-consumption gap may be for a given region, either from a total calorie or nutrition perspective. Being more aware of the gap, and what the gap encompasses, allows for more strategic development of the food system or, if need be, policy for its management during crises. Building on the appraised studies, within the city of Leeds (northern England) an attempt was thus made to empirically quantify its production-consumption self-reliance, with all its nuances, and explore scenarios for lessening (or barriers to lessening) the size and impacts of any gap. Notably, by any measure of size, population or economic activity, Leeds sits somewhere between the Utrecht-10 and Toronto regions and consequently has a well-researched body of food system work to compare and contrast its relative performance with. The following section duly introduces the city of Leeds and the key elements of its food system.

### Leeds and the City food system

**Fig. 1:**
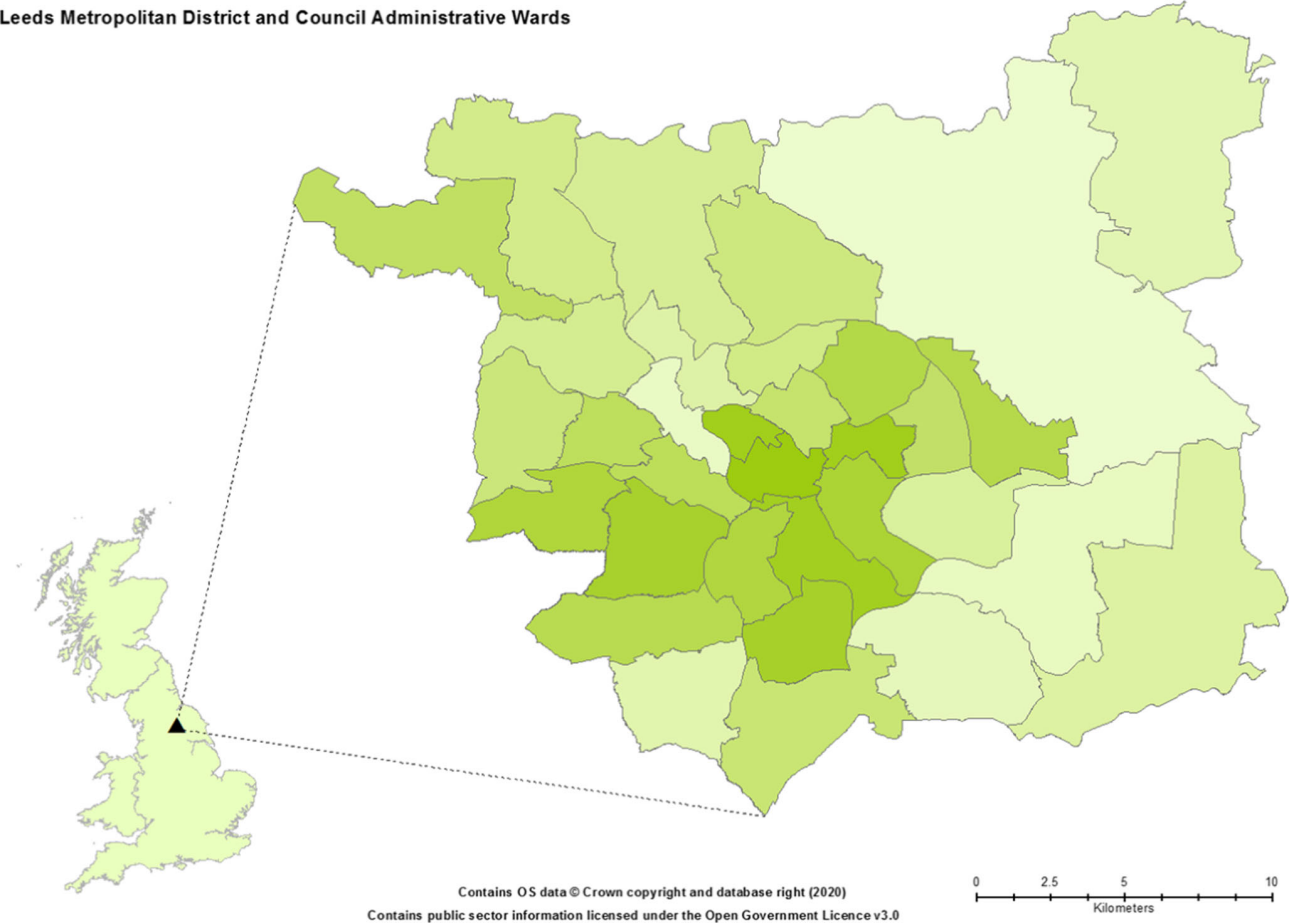
Map of Leeds City Metropolitan Area and Council Administrative Wards. N.B. darker coloured council wards represent greater population density, which are most notably concentrated in the city centre, in contrast to the more rural areas to the east and to the north

As a relatively large English city home to more than 750,000 residents, with a distinct economic centre, peri-urban and rural areas, Leeds is a good example of a modern European metropolitan district (Fig. 1). The metropolitan area sits within a wider formal Leeds City Region encompassing several other cities and large towns within the largest county within the UK, namely Yorkshire.^4^ It is a major public transport and logistics hub and sits at the crossroads of several major highways connected to the rest of world through two of the largest and busiest ports on both sides of the country (i.e., Liverpool and the Mersey in the west, and Kingston upon Hull and the Humber in the east). In terms of local Gross Value Added,^5^ it is the economic centre of the north of England (see ONS 2017),^6^ of which food forms a large element of this economic activity with more than 80 businesses engaged in some form of primary food processing and/or distribution (FSA, 2019). From a human perspective, it is an area of distinct food activity in regard to production and provision and, from a social demographic perspective, possesses distinct spatial trends in respect of wealth, living environment and health, for which all can in many respects be linked back to the performance and properties of the food system and its governance.

**Fig. 2:**
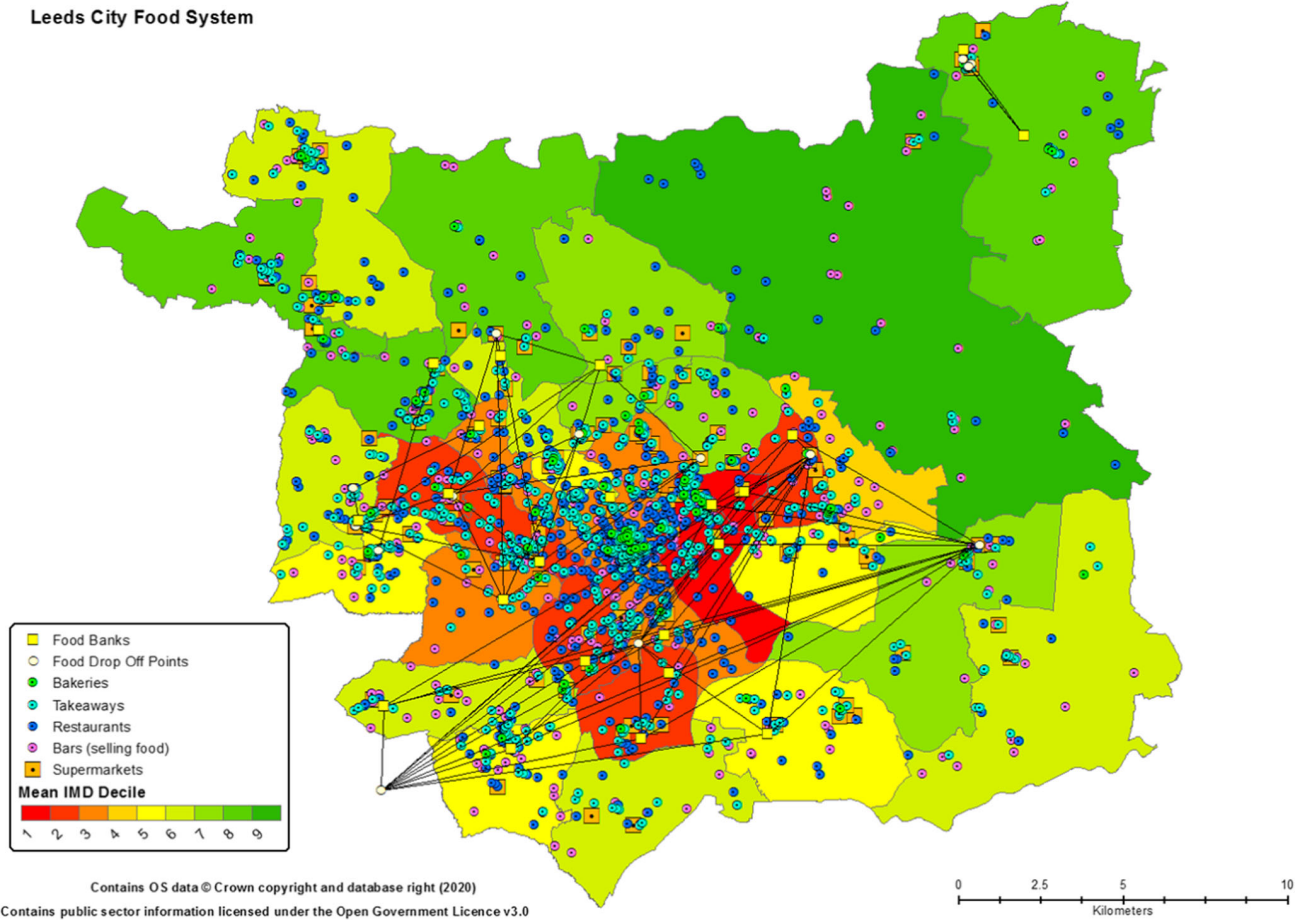
Index of Multiple Deprivation and Number and Range of Leeds Food Outlets. *Note:* the Index of Multiple Deprivation is calculated on a neighbourhood basis but is shown here as an average figure within each council ward as measured in deciles, i.e. red zones equate to an area suffering the highest levels of multiple deprivation relative to the rest of England. Food outlets are largely shown to provide some understanding of the scale and types of food available within the metropolitan area. The lines between food banks highlight their donation collection and distribution network

Despite its prosperity from an economic perspective, the metropolitan area of Leeds shows distinct disparities in the relative deprivation of its residents (Fig. 2). From a food system perspective and its provision of equitable, nutritious food, this is arguably an issue - impoverished communities are adversely affected by a variety of social and environmental justice issues, including food and environmental poverty. Though the UK Government’s official measure of deprivation, an index incorporating aspects of low household income and poor community health and environmental quality, ranks Leeds favourably amongst its fellow northern cities (i.e., Manchester, Liverpool and Kingston upon Hull are all listed in the top five of most deprived cities in England), it is still home to 13 of England’s most “*highly deprived*” neighbourhoods (HCLG 2019: 7). Most of these neighbourhoods are within the heavily populated inner city which is also, as would arguably be expected, the location of the majority of the city’s significant number of food preparation and provision outlets. Indeed, within the metropolitan area there are more than 5500 sources of fresh and prepared food ready for consumption (Fig. 2), ranging from bars that provide cooked meals through to ‘fast food’ providers, restaurants and supermarkets. Sitting alongside this significant food activity and reflecting the high spatial disparity in levels of deprivation, the inner city area is perhaps paradoxically also the location of an extensive food poverty support scheme consisting of 23 ‘food banks’ aimed at providing free food to those most in need.^7^ Demand on this network and similar schemes assisting particular community groupings, has proved significant and grown annually. Indeed, between 2017/18–2018/19, the number of people receiving food from food banks rose 21% to 33,645; while over the same period the number of ready meals provided by drop in centres and street outreach services rose 28% to 104,074 (FAN 2019).

**Fig. 3:**
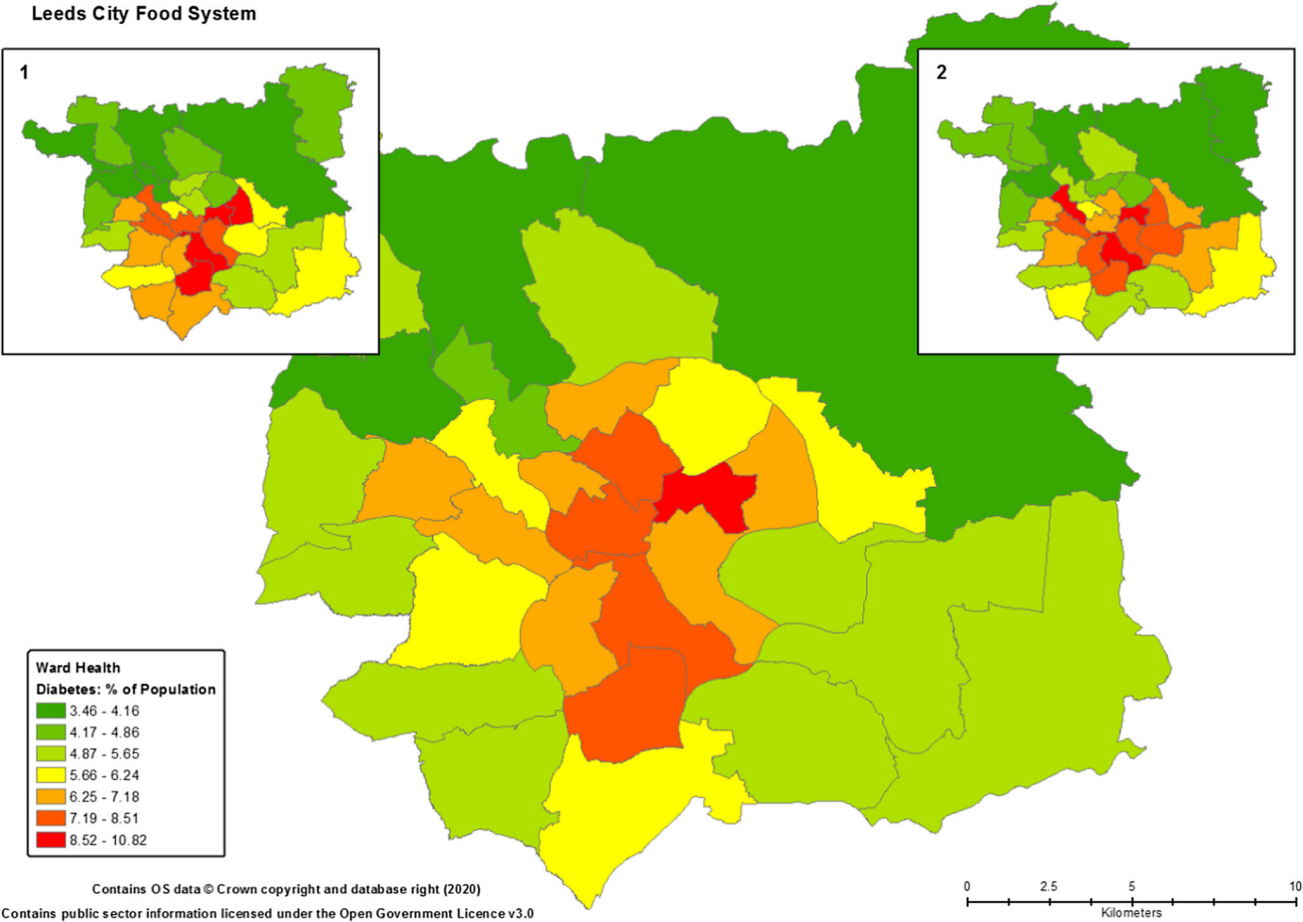
Example of Food Related Health Outcomes within Leeds. *Note:* the main map shows rates of diabetes within the city and its predomination within the more deprived areas (i.e. Fig. 2). Inset map 1 (L) and 2 (R) show rates of coronary heart disease (red zones = 4.6 – 5.1%) and childhood obesity (red zones = 25.7 – 29.8%), respectively (see: NCMP 2018; LCC 2019)

Beyond the presence and number of food support schemes, the city wide disparities in indicators of deprivation are also seen within the health outcomes of residents. Notably, though the rate of adult obesity within Leeds is within the national average (28.7%), and is seen throughout the metropolitan district, the spatial distribution of concerning or chronic health conditions within the city generally follows a similar pattern as that of deprivation. As shown in Fig. 3, the more deprived areas are seemingly those that also possess the greater rates of childhood obesity, coronary heart disease and diabetes (LCC 2019). The prevalence of these health concerns, all possess a relationship to a person’s diet. In respect of the ability of the Leeds food system being able to provide a nutritious and healthy diet to its residents, this is more concerning when it is recognised that the 29.8% upper figure of 10–11 year olds who are formally recognised as obese is almost 10% higher than the national average, whilst the higher rates of CHD and diabetes are both approximately double national averages of 3% and 6%, respectively. Given the current prevalence of such diet related issues within the city, it is perhaps not surprising that past research indicated that less than a quarter (23%) of all residents were believed to consume the recommended five fruit and vegetables a day with modelled estimates of adult ‘five-a-day’ consumption dropping in some areas to less than 10% (HLP 2006). The apparent spatial nexus of prosperity, deprivation, significant food availability, food poverty and related poor health, highlights dysfunctionality and inequalities within the Leeds food system and/or the wider socioeconomic development policies that contribute to its form and function.

Given the above described characteristics of Leeds, good and bad, it can be considered an excellent case study for exploring a modern urban food system, its relative resilience and options for improving food and nutrition equitability and security. A greater understanding of the Leeds urban food system, including physical, environmental and human interactions, could lead to strategies and local policy that allow it and other cities to feed its citizens healthily, sustainably and in a socially just way. The originality of this article lays in its empirical assessment of the consumption-production gap within a modern city, both in respect of absolute energy demand and macronutrient requirements. Such an assessment is missing in many studies but is essential for wider food system activities as it provides the baseline for exploring sustainable strategies for improving food equity and security within the city. Moreover, much of the study was undertaken through extensive data visualisation and spatial analysis within a Geographic Information System (GIS). The use of data visualisations, such as the production of maps made within a GIS, is known to engage more ‘consumers’ of data than text alone (Robinson 2015). Indeed, making the growing amount of interrelated data that is available to researchers for assessment of systems and decision making more accessible is distinct benefit of mapping and spatial analysis of map features. For a regularly community focussed endeavour such as development of urban farming systems, making data that could highlight limitations and drivers of opportunities more accessible to stakeholders and policy makers is crucial.

This article thus focuses on the food supply and demand aspects of the Leeds food system study whilst providing insight into exploratory work into options for improving its performance from a sustainable development perspective. Herein, the article continues by describing the methods employed to map the Leeds food system and to determine whether the city of Leeds could, if necessary, feed itself in an equitable and nutritious manner. The results of the assessment are presented as a discussion in the context of Leeds self-reliance and options for optimising the system in a resource optimised circular economy based manner. The article concludes by contextualising key findings within wider food system study activities, policy development and by providing recommendations for future research.

## Methods

### Data identification and mapping

The spatial mapping and assessment of the Leeds food system was undertaken using publically available datasets whose content was ameliorated, where pertinent, by expert local stakeholder insight. This was largely done to understand what information is freely accessible in regard to food systems, how current and reliable the data is, how such information can be used to assess and perhaps steer the system toward a preferred path, and to assist the replication of the work in regard to future efforts to compare, contrast and determine the typicality of the Leeds food system.

Accordingly, an online search of all government open data websites was conducted with the aim of identifying all information relating to Leeds and the primary aspects of the food system that were identified, through author and stakeholder discussion, to be of relevance or important within a sustainable and resilient food system, i.e., technical, socioeconomic, environmental indicators of food system facilitation or performance. Given the desire to visualise and spatially explore relationships between food system components, the search was optimised in respect of identifying sources of information that possessed a spatial reference that allowed the data to be mapped or assessed at a higher resolution than the city metropolitan boundary, i.e. local council wards, postal codes or smaller census statistical units. Via the UK government’s ‘Open Data’ website, data from the Office for National Statistics (ONS), Public Health England (PHE), Department for Environment, Food and Rural Affairs (Defra), relating to measures of poverty and demographics, health and wellbeing, and food production, was identified. A further search was performed of the UK’s dedicated mapping agency, the Ordnance Survey (OS), for all vector data available for the Leeds city region’s infrastructure and natural features of relevance or that promote or indeed limit food production, i.e. road networks, buildings, sources of water, flood plains, soil types and land type.

As with all system focussed studies, the choice of performance indicators is driven by context. Based on the long term goals of the work relating to resilient and equitable access to nutritious sustainable locally produced food, it was thus deemed that inter-related indicators relating to diet, health, living environment and food production and provision, would be sought. For example, local rates of obesity and diabetes Type II, which can be directly linked to diet, were sought; as were data showing broad indicators of living environment, food production and food provision. Such data, in this regard, included searches for air quality records, levels of available green space, land cover records, food bank networks and types of food outlets. Much of this data derived from the search of the government’s Open Data website or its links through to other agencies and the local council’s website (LCC) (see Table 1). However, through discussions with stakeholders relating to the nascent food system indicator dataset, other more nuanced information was made available which placed much of the data in context. For instance, detail on the location *and* supply network of food banks was directly provided by contacts. This information was, nonetheless, still available publically, thus maintaining the desire to use, as far as was possible, freely available information.

**Table 1:** Mapped Publically Available Food System Data Employed in Wider Study

**MAPPED CITY REGION FEATURE DATA**		
**Land Character**	**Environment**	**Buildings**
Soil Types Allotments Woodlands Green Spaces Crop Types (2016–2018)	PV Location/Capacity Air Pollution (Automated) Air Pollution (Passive) Fertiliser/Pesticide Use Recycling Bins/Types Recycling Bring Sites Environmental Groups	Residential Glasshouses Educational Sites Derelict Buildings Commercial Buildings Other Functional Sites
**Hydrology**	**Community Food**	**Transport**
Water Supplies Boreholes Surface Water Flood Risk Hydrogeology Land Permeability	**Support** Food Banks Food Bank Drop Offs Rethink Food Healthy Holidays Lunch Clubs (Over 55 s) Community Centres Other Community Facilities	Railway Station Railway Track EV Charging Point Minor Road Network Primary Road Network
**SPATIAL DATA ATTRIBUTED TO COUNCIL WARDS**		
**Demographics**	**Diet and Health**	**Living Environment**
Population (2019): *Total of ward* *Gender distribution* *Age range and distribution* Index of Multiple Deprivation (2019, though contains older indicator data): *Weighted by: Income (22.5%), Employment (22.5%), Education (13.5%), Health (13.5%), Crime (9.3%), Housing Barriers (9.3%), Living Environment (9.3%)*	Percent of ‘Five a Day’ (2010): *adult and children* Childhood Obesity (2013–18): at school reception and Year 6 Adult Obesity (2013–18): mapped as 5 yr mean and 5 yr % change DASR Diabetes (2013–18): mapped as 5 yr mean and 5 yr % change DASR CHD (2013–18): mapped as 5 yr mean and 5 yr % change DASR Smoking (2013–18): mapped as 5 yr mean and 5 yr % change	Building Density: inc. number of pubs, takeaways, restaurants, supermarkets Green Space Density: type of amenity and total hectare Woodland Density: type of woodland and total hectare Air Pollution: annual mean and annual daily mean NO_2_m^3^ Misc. feature/pp. calculations

*Note:* unless indicated otherwise, the mapped data was produced or updated, and assumed correct, in 2019/2020. See reference list and Supplementary Material for all sources of data.

Each of the identified datasets for the key system indicators was duly prepped for import to a GIS system (in this case, ArcMap 10.6). This largely involved placing all relevant and related data, produced at the same spatial scale, into excel files and removing any superfluous information and/or renaming information to formats acceptable to the GIS (e.g., avoiding spaces in titles and non alphanumeric characters were possible). All datasets were then imported to the GIS and overlain onto boundary maps of city council wards, the city metropolitan area, the wider city region and the vector data for infrastructure and natural features sourced from the Ordnance Survey. For data which contained geographic coordinates, this duly resulted in the production of multi-attribute data points for a variety of indicators (e.g., food outlets, allotments). For information not possessing a dedicated geographic coordinate, the data was imported as a table and, using the GIS’s *join by attribute* function, the table’s indicators and all related attributes were joined using the names of the council ward layers. This was repeated for the data-points but, in this instance, the *join by location* function was employed and the sum of each point’s attributes was assigned to the ward layer. For polygon data, such as buildings, green space and land cover, the *calculate geometry* function of the ward layer’s attribute table was accordingly used to calculate the total area of each indicator.

Following the spatial joining of data, the mapped Leeds council wards possessed data on a variety of food system indicators relating to the performance of the city’s food system and wider functioning in regard to the health and wellbeing of its residents. The populated attribute table of the base map and overlaying feature data was processed into a variety of choropleth, bi/multivariate and spatial distribution formats to visualise the performance of the food system and relationships between indicators, including population density, deprivation, health and types and density of food outlets, their proximity to public building such as schools, and, most relevant to this study, the amount and types of food being produced in the city and its peri-urban and urban areas.

Wherever possible, insight produced by the GIS was cross-referenced for accuracy with other data. For example, for the land cover maps that provided the location, area and type of crops being produced, this was cross-referenced with Defra annual crop production returns for the city region (i.e., Defra 2019). Notably, however, the public crop production information provided by Defra omits, for a variety of technical and sometimes anonymity reasons, the tonnage of several crops. By cross-referencing this data with the land cover map layer, produced using remote sensing, it was possible to estimate the amount of land a given crop was being grown on in a given area and reliably estimate,^8^ based on tallied totals within the farm returns, the potential yield of all crops over a growing season. As not detectable by the mapping process, animal counts in the region were directly derived from the national farm accounts along with a figure for milk production and total number of laying eggs. For the number of hens directly employed in laying, a typical annual egg laying figure was employed (i.e., 290).

### Calculating nutrient production and demand

From the duly calculated annual tonnages of food production within the city region boundaries, a figure for average usable energy and nutrient content for each known crop and animal product was sought (e.g., for wheat, only the metabolisable energy content of the grain was calculated, and for meat products, tonnages were reduced by the relevant ‘killing out’ proportion per head of stock). To provide nuance to the determination of any gap in food production and demand, the macronutrient content of the assessed range of locally produced foods was also calculated (micronutrient content remains work in progress within complimentary health focussed studies). To determine food demand in the region, the population figures, gender ratio and age profile for each council ward, provided within mapped deprivation data, was aggregated into three age groups. For the age groupings, namely 0–15, 16–64 and 65+, the specific daily food demand (in kcals) for age and gender, within the three groups, was ascertained using the guidance provided within the UK Government’s most recent ‘Dietary Reference Values for Energy’ (SACN 2011). The sum figure for each grouping was then aggregated to produce a total energy and macronutrient demand for the city region. Notably, after producing the respective demand figures for each age group, it was decided to revert to using the widely used and recommended average daily calorie intake of 2500 kcal and 2000 kcal for the 16 to 64 age group. This decision was made due to UK Government guidelines for higher food demand in the 16–64 range being based on the increasing size of individuals (on a trajectory toward obesity), rather than typical dietary need. From a food systems perspective, where sustainability, security and indeed food equitability is a distinct consideration, the former value did not seem an appropriate figure to use. As such, it should be noted that the figures presented within the results for calorie demand, could be 52.4billion higher. This highlights a distinct point of additional discussion, outside the scope of this article, around ‘desire’ and ‘need’ in regard to food demand, which can be a highly subjective and nuanced indicator to be managed within food systems.

## Results and discussion

### Overview of the wider region

**Fig. 4:**
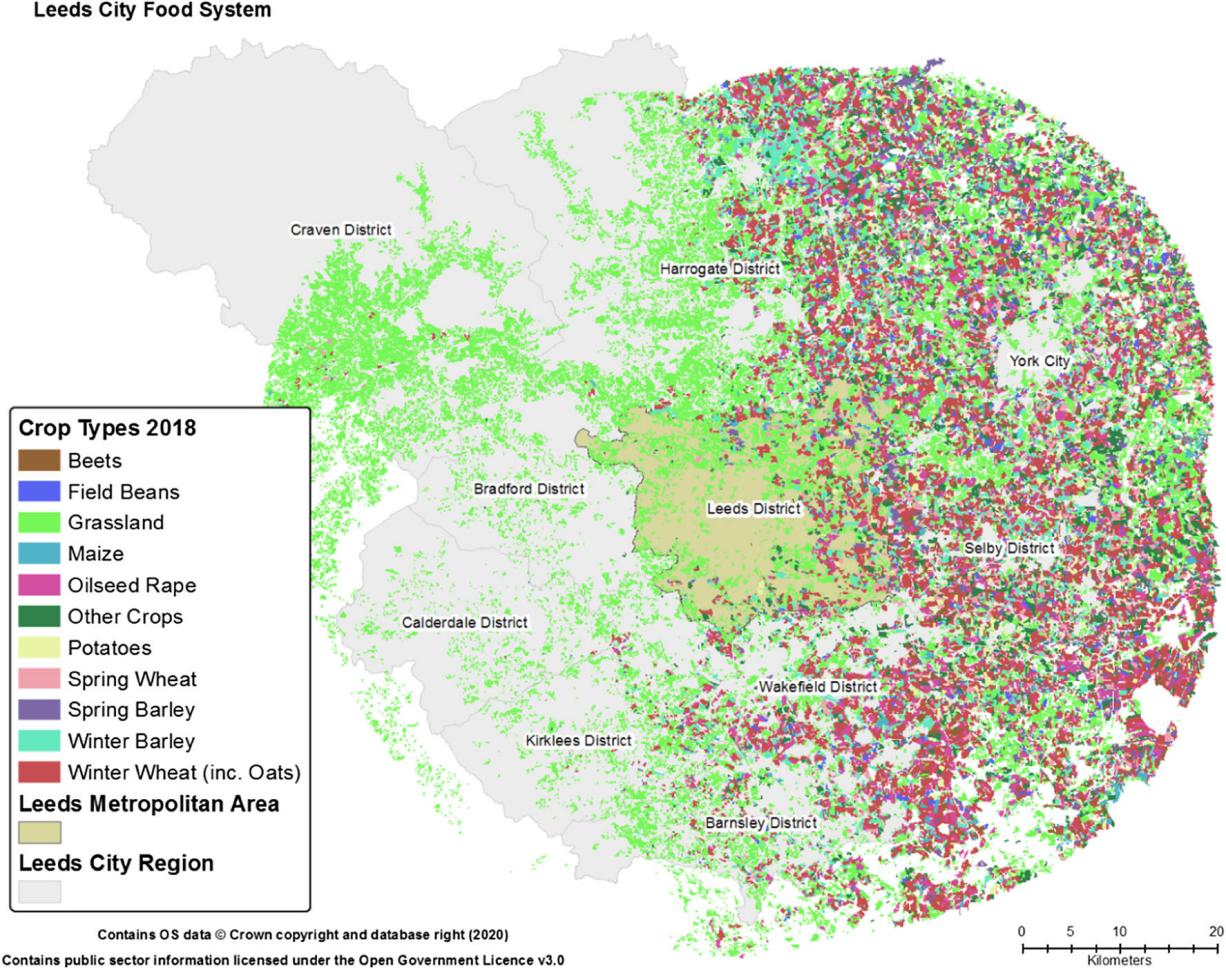
Leeds City Region and Crop Type Production in the Surrounding Area. *Note:* at the scale depicted it is difficult to differentiate land parcels, the map is primarily provided to emphasis spatial variation in regional crop production and potential availability

Though the focus of this paper is the Leeds metropolitan area, in respect of ongoing research into potentially optimising the efficiency of the system and understanding opportunities offered by the wider city region (discussed below), the immediate mapping area beyond the metropolitan boundary of Leeds was initially set at 33 km as this distance has been shown to be important in the development of regional resource efficiency (e.g., Jensen et al. 2011; Jensen 2016). The regional data mapping process highlighted that Leeds and the wider city region is an area of distinct food system activity; albeit, as shown in Fig. 4, there are clear geographic differences in activities across the extended region’s 5720km^2^. Indeed, within the mapped 33 km zone, it can be seen that agricultural production is largely concentrated to the east of the region with the west, aside from some areas of grassland employed in livestock grazing, largely devoid of primary crop production. Indeed, grasslands, whether permanent or rotationally used for grazing and leys, make up the majority of ‘production’ in the area. Using the *Calculate Geometry* function of the GIS to derive the total area of crops within the land cover data, this observation could be quantified. Duly, it was found that from 2016 to 2018, grassland made up 41% of the approximate 341,000 ha (Ha) of total parcels of agricultural land within the 33 km zone, with growth of spring and winter wheat being the next most prominent use of agricultural land (23%). In regard to these agricultural variations, it should be noted that the area to the west, which appears largely devoid of agricultural activity, contains other relatively large urban conurbations, most notably, population wise, the Bradford metropolitan district (with ~540,000 residents) and Kirklees (incorporating the large market town of Huddersfield, with ~440,000 residents).

### Production in the City boundary

**Fig. 5:**
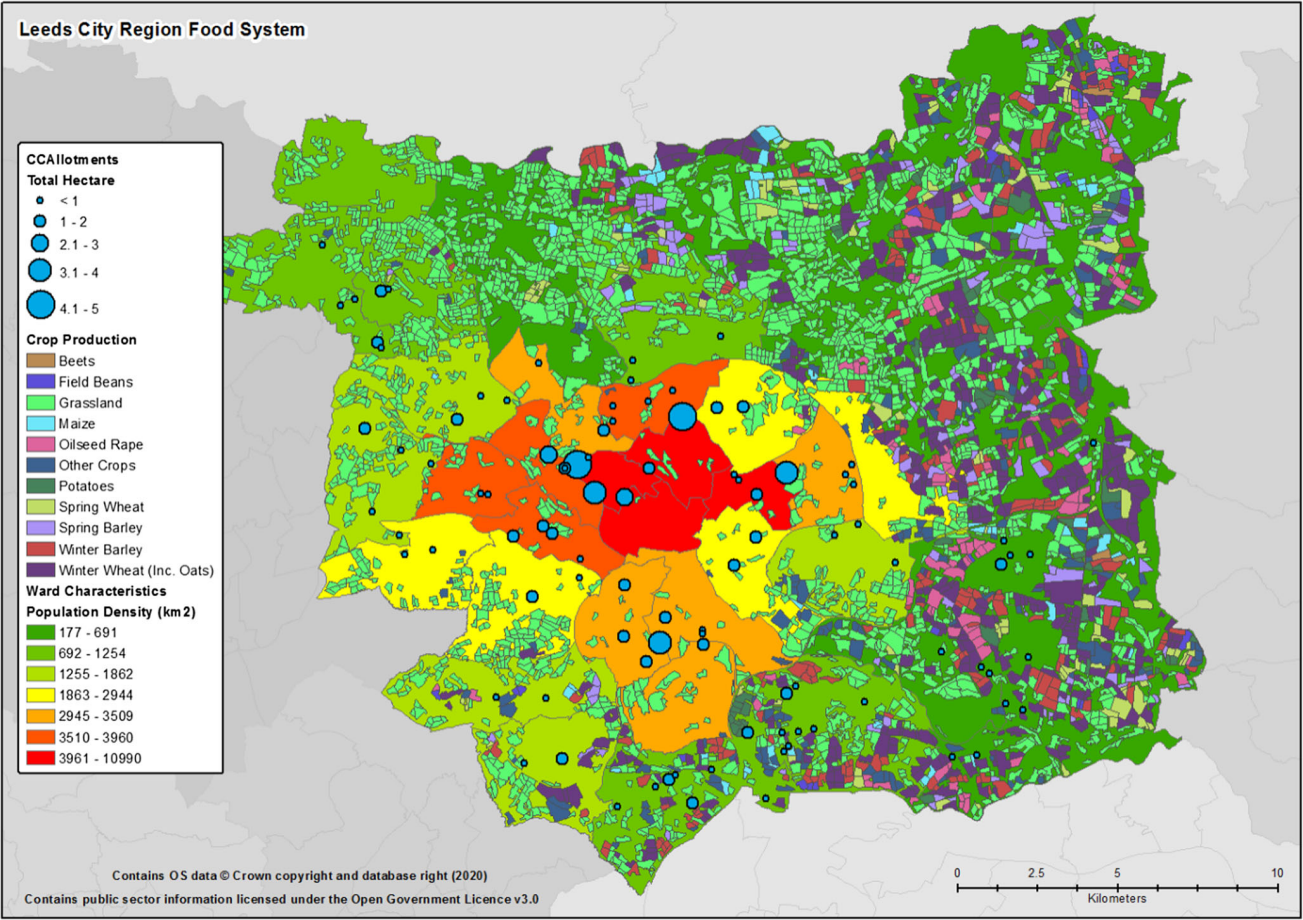
Agricultural Land Use and Type in Leeds City Metropolitan Area

Focusing on the metropolitan district of Leeds, which includes the city and the immediate urban and rural areas within the control of the local authority (as depicted in Fig. 5), the mapping exercise and cross reference to the anonymised agricultural production tables, indicated that the district’s ~ 23,000Ha of agricultural land produced almost 300billion kilocalories (291.6B kcal) of metabolisable food in 2018 for consumption by humans (i.e., the figure excludes feed/energy crops and nursery/breeding stock). The vast majority of the produced energy derived from arable products and the edible content of wheat and barley grain (~131B kcal, ~80B kcal, respectively). The largest share of animal derived products (92%) was found to be in the form of milk production and beef (both ~9B kcal) (see Table 2). Time series land cover maps highlighted that crop rotation and changes to grazing area does occur on an annual basis, however the changes in total area and crops being produced was negligible for the years that could be mapped (i.e. 2016–2018), with most variation existing between types of grain production. As such, with minimal variation in area cultivated and crops grown, it is assumed that the total figure calculated for metabolisable food production is typical and applicable over the most recent past and immediate future.

**Table 2:** Summary of Dietary Content of Food Produced for Human Consumption within Leeds

**Food Type**	**PRODUCTION**			
	**Total Calories** **(Billion kcal)**	**Protein** **(kt)**	**Fat** **(kt)**	**Available Carbohydrates**^**1**^ **(kt)**
Plant Based Products	272.3	8.5	5.0	38.6
Animal Derived Products	19.3	1.6	1.2	0.6
**Total Production**	**291.6**	**10.1**	**6.2**	**39.2**

*Note:* the estimation of available energy and macronutrients represents the edible mass of the food types following initial processing, e.g. minus cereal husks, shells or ‘killed-out’ proportion of an animal carcass. ^1^Available Carbohydrates are broadly defined as starch and sugars and do *not* include dietary fibre (FAO 1998).

As noted throughout this study, within all populated areas there is a need to provide a varied and healthy diet to people of all ages and needs. The above production assessment largely focussed on energy production in the form of total calories. Though this would be important in the short term and in response to the immediate impacts of any scenario where greater dependence on local food supplies may arise, it does not cater for medium to long term nutritional needs. Further work is needed to determine the specific micronutrients provided within the ~300billion kcal of crops and animal products produced within Leeds; however, the lack of crop diversity would suggest that sufficient amounts of micronutrients and omega 3 fatty acids, for example, are unlikely to be produced from an agricultural base in the north of England currently dominated by cereals, potatoes and food products derived from cows (i.e., milk and beef). However, with cross-reference to a food composition database, it is it is relatively simple to derive an estimate of the macronutrient content of the food produced in Leeds. Based on the calculated tonnage of crops and animal products it was be duly estimated that 10.1kt of protein, 6.2kt of fat, 39.2kt of *net* or available carbohydrates and 9.1kt of dietary fibre is typically produced within the city in recent years (i.e., 2016–2018). When combined, the available macronutrient tonnage, including fibre, equates to approximately 58% of the total mass of all locally produced foodstuffs, with the balance made up of varying proportions of water, ash and minor organic compounds (e.g., vitamins and nucleic acids).

### Food demand in the City

As part of the food equitability aspects of the food system mapping project, the UK Government’s Multiple Index of Deprivation statistics, assigned to Lower Super Output Areas, were mapped onto the council ward boundaries which they fell within and aggregated or averaged depending on the type of data^9^ (i.e., population figures were summed, indices were averaged). From this exercise the total population for each ward and the age and gender profiles were derived, with the assessment indicating that 784,846 people live within the metropolitan area, largely within the inner-city. The age profiling exercise showed that of the total population 65.3% are of working age (i.e., 16–64) and live predominantly within the inner-city highly urbanised areas; 19.2% are infants and children (i.e., 0–15) and 15.5% were 65 or older, many of which, lived within the suburbs or rural areas of the metropolitan boundary. As described within Section 3, based on mean gender calorie and macronutrient demands for each age range (calculated to be 1638 kcal across 0–4, 5–10, 10-15 yrs.; 2245 for 16-64 yrs.; 2093 kcal for 65>), it was determined that the total demand of the city was more than 600billion kcal (603,011,707,839) per annum. Based on Public Health England’s most recent dietary recommendations (PHE 2016), it was further calculated that, based on the city’s population and age profile, the city had an annual macronutrient demand of ~13.1.kt of protein, ~23.6kt of fat and ~ 79.6kt of available carbohydrates (i.e., *not* including dietary fibre, which would equate to ~7.9kt for the age profile of Leeds population) (Table 3).

**Table 3:** Nutrition Demand of Leeds City Residents

**POPULATION**			**AGE RANGE AND GENDER CORRECTED NUTITIONAL DEMAND**			
**Age Range**	**M/F** **Ratio**	**Number**	**Calories** **(kcal)**	**Protein** **(kt)**	**Fat** **(kt)**	**Available Carbohydrates (kt)**
0–15	49:51	150,447	89,934,372,177	1.47	3.64	11.15
16–64		512,798	420,199,501,150	9.37	16.34	56.02
≥65		121,601	92,877,834,512	2.21	3.60	12.37
**Total**		**784,846**	**603,011,707,839**	**13.05**	**23.59**	**79.55**
**Potential Local Supply (%)**^**1**^			**48.4**	**77.6**	**26.1**	**49.3**

*Note:* calculated figures for nutritional demand are based on UK government age dependant dietary guidelines (see SACN 2011). ^1.^ Based on production figures shown in Table 2.

### Rationalising and closing the gap

From the above it can be seen that that the gap between food production and demand in Leeds is in the region of 310billion kcal (i.e., ~603billion – ~291.6billion). In overarching terms, this translates as the city being 48.4% self-sufficient or, in respect of metabolisable energy provision, possessing a 51.6% deficiency in year on year food security and resilience. This, notably, is in line with a UK production-consumption gap that, minus minimal exports, stands at 50% (Defra 2018). In respect of macronutrient supply and provision of a sufficiently nutritious diet, the deficit is more striking. Analysis of nutrient production in Section 3.2 demonstrates that available carbohydrate dominates production (i.e., 39.2kt, or 61% of total nutrient tonnage), but does not meet the demands of the city. Protein and fat production, meanwhile, demonstrate extremes in deficits. Though both are produced in minimal amounts compared to available carbohydrates, the deficit in supply is 22.4% and 73.9% for protein and fat respectively. Beyond a probable shortage in locally produced micronutrients discussed in Section 3.3, this analysis suggests that any targeted attempts to increase nutrition availability within the local food system would need to pay particular consideration to the provision of (healthy) fats.

In respect of any arguments for wanting or needing to close this gap, referring back to Fig. 4, it can be seen that simply extending the boundaries of what is deemed the local food system would not necessarily provide a solution to the lack of regional food security. Indeed, Fig. 4 shows that the west of the wider city region is heavily urbanised with several other prominent cities who could lay claim to any locally produced resources. Meanwhile, the area to the east, beyond the city’s metropolitan boundary, lies largely within other planning and council authorities control and, in terms of any form of crisis management, other Local Resilience Forums.^10^ As such, any considerations around improving the resilience of the city in respect of its ability to feed its population, in a crisis situation or otherwise, would have to consider these points. That is to say, if such a situation did arise where the city was reliant on local resources for any period of time, any planned or ad-hoc solution would not be as simple as looking directly beyond the city’s boundaries for a reliable supply of nutritious food, particularly as the wider country can only meet 50% of its demand for food from current native production.

Moreover, as has been noted elsewhere (Defra 2009), food security and resilience is different to, and goes beyond, being self-sufficient. Further exploring this premise and the feasibility of the city to sustainably grow more food, based on a total metropolitan area of 55,172 ha, and a population of 784,846, there is 0.07Ha/pp. directly, ‘on the ground’, land available for food production. This figure, based on an impossible scenario of every square metre of land within the city and urban area being cultivated, falls some way behind the FAO (2011a, b) estimation that a minimum of 0.22Ha/pp. is required to adequately feed an individual for a year,^11^ and is commensurate with similar findings for the neighbouring area of Kirklees (i.e., Lever et al. 2016). the Leeds Ha/pp. is also, notably, less than the average 0.09Ha/pp. available to the wider population of the UK which, due to its population density, is itself some way behind the European Union’s requisite 0.22 (i.e., World Bank 2020). Even if extending its boundary was possible, such figures for the UK suggest that this would not necessarily provide an immediate solution to any food security concerns. Moreover, simply extending boundaries does not fit in with the idea of ‘local production’, which, within urban agriculture studies, has been acknowledged to be a subjective term (Morrison et al. 2011). Moreover, the urban food system idea is connected to: *“so-called “zero miles” food production*” (Castrica et al. 2020: 2). As such, zero miles production is, where possible, something that from a city resilience and environmental perspective should be aspired to, as it implies that the city is closer to being sustainable and self-reliant. The mapping and wider data appraisal of the Leeds food system, however, suggest that growing options within the city in regard to closing the demand and resilience gap are currently minimal.

Though small niche growing activities are taking place or planned within the city (e.g., community interest farming groups), no industrial scale vertical farming or similar urban growing innovations were found to be taking place within the city that could notably compliment or add to the production of food in traditional production settings. As shown within Fig. 5, however, it can be seen that parcels of allotments ranging from <1Ha to 5Ha are distributed throughout the inner-city. Again using the *Calculate Geometry* function of the GIS, tallied to council and private allotment records, it was possible to estimate the scale of these urban growing areas. It was found that the largest allotments where located within the densely populated areas of the city of which 93Ha in total were under council control and complimented by a further ~39Ha of allotment and community growing areas within private hands. This compares favourably to the reported 97Ha within Leicester and, when including the privately owned allotments, nearby Sheffield’s 138Ha. Based on Edmondson et al. (2020a, 2020b) potential production figures of up to 1.8 kg/m^−2^/yr^−1^ of fruits and vegetables on allotments in the UK, this would suggest that almost 2400 t of mixed horticultural produce could potentially be cultivated within the city per annum. This, of course, assumes that all allotment area would be in use (i.e. it excludes the almost certain presence of access paths and storage areas) and that all growers are horticulturally adept. Nutrition wise, though such use of allotments producing additional fruits and vegetables could contribute to micronutrient demands, such a potential total production figure, even if solely growing one of the more easily cultivated and calorie dense crops such as potato, could only add in the region of 2–4 billion kcal,^12^ i.e. closing the production-demand gap by 0.3–0.6%. Additionally, any coordinated attempt to bring allotments into a wider city food provision scenario, questions could arise over the delegation and governance of managing such dispersed multiply stewarded land.

**Fig. 6:**
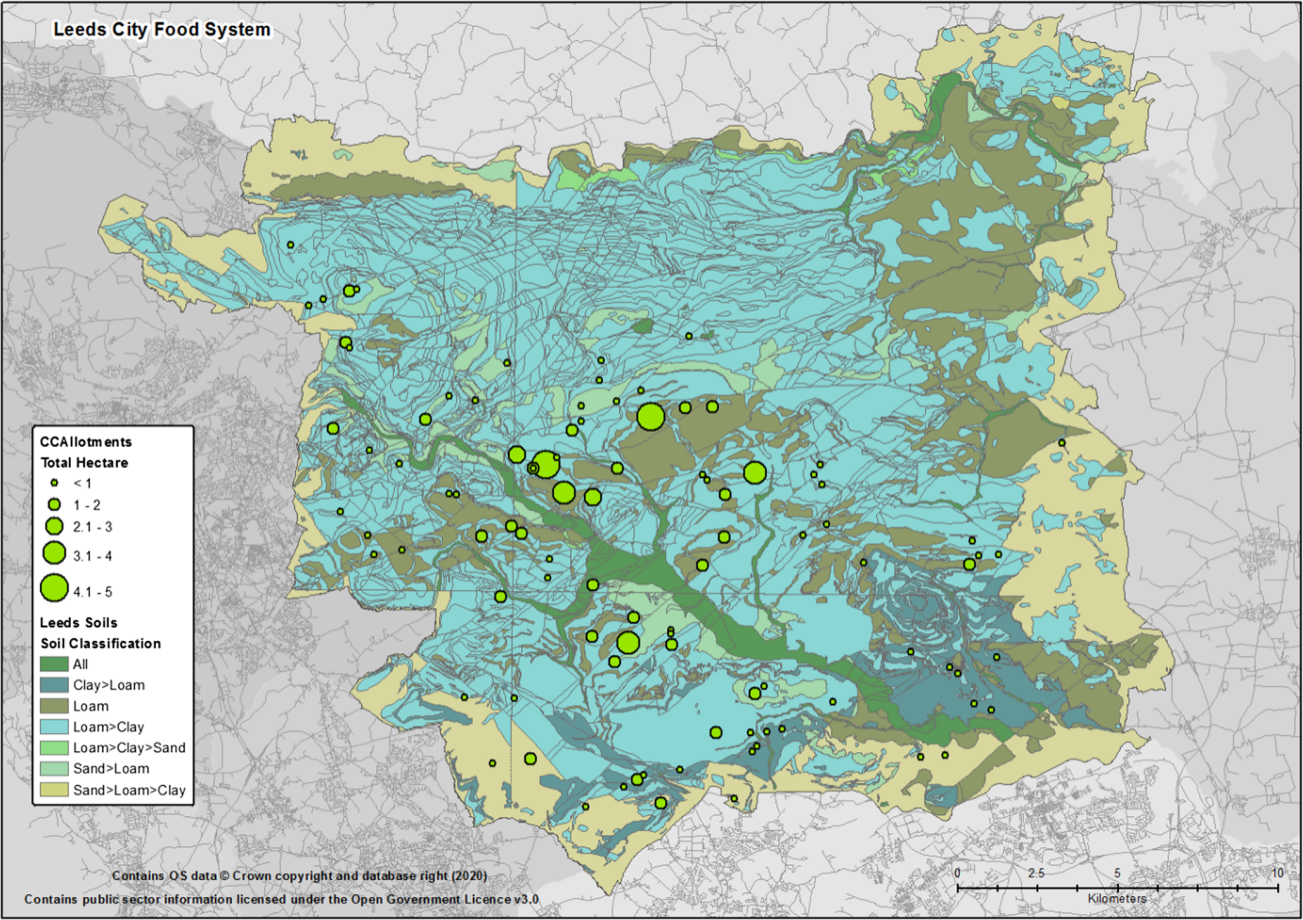
Leeds City Soil Type Distribution and Surrounding City Region Urban Districts

Moreover, as a product of a carefully balanced ecological system, food production is of course more than a matter of available growing area. Whether by historic accident or design it is noticeable from the mapping process that Leeds allotments, and much of Leeds more diverse areas of industrial farmland, lay on the better loamy soils away from the primary flood plains (Fig. 6). This suggests that arguments for boosting local food security through repurposing of existing green spaces and gardens (e.g., Edmondson et al. 2020b), though possible, would not necessarily be achievable in Leeds without significant access to labour and/or imports of growing material and other necessary resources.^13^ This observation highlights one of the benefits of the food system mapping and visualisation process and the value of mapping all potential indicators of the system’s wider operation and function. For such repurposing of city assets, such as allotments, greenspace and other spaces and infrastructure, careful assessment of the wider community impacts would be required if the sustainability of urban agriculture is to be assured. Indeed, though UK councils are obliged to provide urban residents with sufficient growing space, from a social perspective it has to be recognised that existing green spaces are intrinsically valuable to residents’ well-being - albeit other studies have raised the potential of urban agriculture as a way of reconnecting people with nature and food or, simply, as a pleasurable viewing experience (Wiltshire 2010). Overall, it has been noted that urban agriculture, in its variety of forms, must be developed in a careful manner to make best use of available ecosystem services whilst not transferring environmental impacts from one place to another (e.g., Russo and Cirella 2019; Beacham et al. 2019). Any marked increase in local production of nutritious foods would thus require some level of innovation, community coordination and associated investment.

### Potential urban food system innovation

Having considered the expansion of the Leeds food system boundary and increasing local food production in more conventional ways through greater use of green space, the opportunity to increase nutrition security and equitability could exist through system optimisation, including multi-spatial and temporal use of available growing areas and resources. As demonstrated within the introduction to Leeds, it is home to a significant amount of food activity, including food outlets that produce waste and food support ‘banks’ that redistribute excess or donated food from a variety of sources. The presence of such an established network of food outlets and food banks, along with other underutilised or abandoned city infrastructure, could help bring food production innovation into the city by actively pursuing a circular economy based urban agriculture that, to some extent, could limit the resource constraints and contention that possibly emanate from the repurposing of a city’s green space. It is known that vertical farming and similar innovative uses of space within urban areas can be energy and resource intensive (Jenkins 2018; Beacham et al. 2019). However, this is where the benefits of a circular economy based food system could be witnessed, particularly with regard to utilities sharing and the efficient use and reuse of water and many essential nutrients. Going beyond simple recycling of products, a circular economy is designed to conserve and regenerate resources within a system and can and should do so in an environmentally and socioeconomically just manner (Velenturf et al. 2019). Indeed, circular economy simply embodies the practices and long held principles of agroecology.

Detailed discussions on the technical development of a circular economy based food system within Leeds and the socioeconomic and environmental benefits of such a system are beyond the scope this article. However, the mapping process has highlighted the scale of food based activities that are taking place within Leeds and the spatial (food and health) inequalities that also exist that could perhaps benefit from a circular city-based food ecosystem. Indeed, notably, the Utrecht-10 RUAF study considered the role of a circular economy in their city-region and the beneficial effect it could have on the system, including in respect of development of local business opportunities (Haenen et al. 2018). However, beyond exploratory research, at the time of writing they reported limited signs of a circular economy being connected to the region’s food system – this was true for innovative valorisation of food wastes to produce essential nutrients or, more simply, in the areas of energy (re)use or conservation. It was suggested that, much of this limited activity could be due to economies of scale and/or existing restrictive policy and legislation, i.e. unintended barriers created by existing narrowly focussed system governance. Though there are particular policy issues with (re)use of biological wastes, policy in numerous regions is, however, changing toward adoption of circular economy (UNIDO 2017; EC 2020) and, within a non-profit community based food system, the particular barrier of scale is or could be somewhat lessened.

A cursory spatial analysis of options for reorganising urban assets able to engage in a circular food system showed that there are 518km^2^ of warehousing in the centre of Leeds in various stages of occupancy, with direct or possible connections to renewable energy and water, that could be employed in, for example, significant vertical farming efforts and/or other innovative green wall or rooftop agriculture. Perhaps of more immediate and obvious use, however, there is 200km^2^ (20,000Ha) of derelict or vacant buildings and land with a total of 544km^2^ of floor space within the city that also lay within areas of notable renewable energy production (or opportunities) and in close proximity to food banks, community centres and numerous food processors and outlets (i.e. potential ready sources of nutrient dense wastes, growing medium and containers). Though there may be questions over the ability to use the entire floor space of buildings and land classed as derelict (e.g., due to concerns over building integrity or land contamination), they represent underutilised city assets that could be incorporated into a resource efficient food ecosystem within the auspices of innovative vertical farming, hydroponics and/or through more conventional, low or no dig methods (perhaps within containers where concerns over land or building integrity exist). Most notably, many of these areas of potential food system symbiosis are found to be within those suffering most from food poverty, diet related health issues and a limited intake of fruit and vegetables, i.e. those who are usually the first to suffer during a crisis situation.

**Fig. 7:**
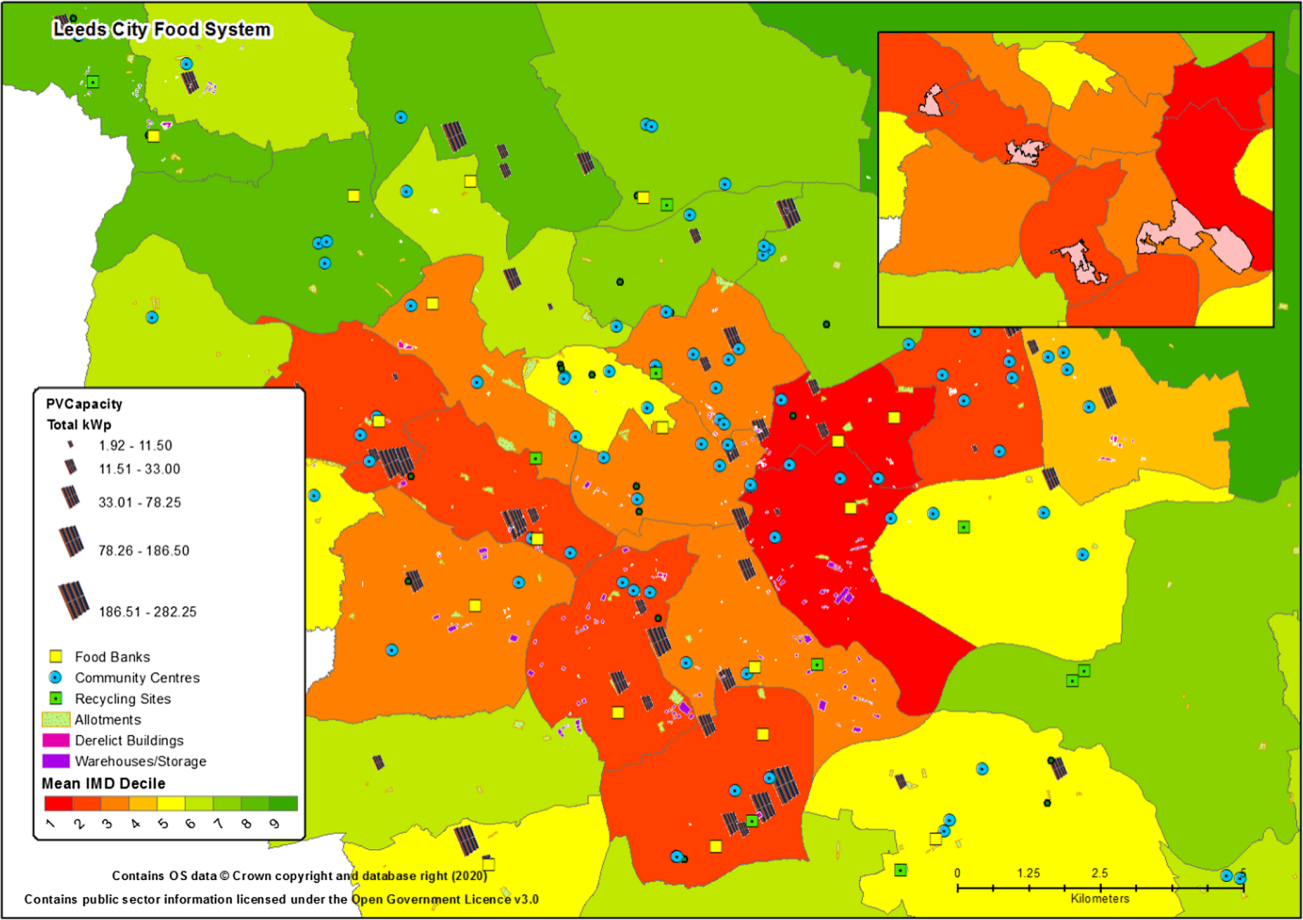
Urban Agriculture Opportunities and Potential Food Hubs. *Note:* at the scale shown it is difficult to differentiate between depicted food system assets and other key assets, such as water availability and access points, green space and trees (e.g., pollinator havens). Figure 7 is primarily shown to depict the number of food system assets that exist within the close proximity of community food banks and options that exist for potentially creating circular locally resourced food production, processing and provision food hubs. The inset image highlights neighbourhoods that contain a large concentration of food system assets in close proximity to each other and potential locations for community food hubs

Creating a symbiosis between communities officially classified as multiply deprived, underutilised local assets and infrastructure, and the activities of those operating within the local food sector that are potential sources of critical resources, presents opportunities for myriad beneficial food production, processing, distribution and education hubs (Fig. 7). Though studies on the subject are currently limited, as stated elsewhere within work on urban vertical hydroponics, resource symbiotic circular production systems have and could indeed improve the environmental performance of such systems whilst providing wider learning and social development benefits (Martin et al. 2019). As highlighted above, such innovative options for community production could be an option for Leeds or similar cities.^14^ Though extremely unlikely to provide the total calories or macronutrients required to lessen the city’s production-consumption gap and thus notably increase its absolute food security, community food hubs existing within a local circular economy could provide those most in need with an appreciable level of microsalads or fungi that are a source of many micronutrients and, perhaps, the protein and some fats that the city is deficient in (i.e. through farmed fish^15^). Such endeavours would, however, face the same sustained engagement, access to labour and coordination challenges as any other proposal for community farming. Moreover, the development of a symbiotic or smart urban food system, requiring the matching and integration of practices and social and technical assets, would require critical appraisal of urban food governance in all its forms, including the production, collection and provision of food system data (e.g., Maye 2018; Helenius et al. 2020). Beyond the long-term encouragement of bringing local food onto local plates through lobbying of local producers and supermarkets, and thus reducing the reliance on precarious global supply chains, an urban food symbiosis within a circular economy would seem however to be one of the better options for improving the city’s food resilience in a socioeconomically and environmentally sound manner, albeit still likely to leave the city someway short of ever being self-reliant.^16^

### Limitations to results

The total calorie production figure could be affected by a number of uncertainties, for example within the continued production of crop types over a series of growing seasons and/or within the accuracy of the remote sensing techniques used to determine crops being grown within the Land Cover maps. However, the producer of the Land Cover data, The Centre for Ecology and Hydrology, indicate a crop identification accuracy of 90%, 95% and 97% for cereals, improved grass and oilseed rape, respectively; with beans and root crops returning an accuracy of 80% (see CEH 2018). Given the predominance of grass and cereal production within the region, and the similarity of total crop hectares provided within the Defra regional farm output database, some confidence can be placed in the calculated city-wide production totals. Moreover, in contrast to many food system and urban agriculture studies, which employ broad estimates of food availability based on total populations and production tonnages, within this article an attempt has been made, wherever possible, to determine calorie figures from the specific edible and killing out portions of the region’s produce. As such, any missing calories that may have been lost in the Land Cover data or Defra reference accounts, or within other unidentified urban agriculture activities, are compensated for with the accuracy of final figures in regard to edible content of foods and indeed the use of a specific age and gender calorie demand profile for the city, instead of a generic daily kcal/pp. figure. Additionally, in respect of the demand figure, as highlighted within Section 2, the denominator employed could also be higher if the higher national dietary guidelines were employed (i.e. the demand figure was reduced by 52Billion kcal to avoid obesity). It also must be recognised that it is unlikely that 100% of the useable portion of the food produced in the area could be consumed. Invariably, there is food wastage at all stages of the supply chain during food processing and during preparation, whilst some spoiling and waste is always present within food systems. As such, it is with some confidence that the food production-demand deficit for Leeds is presented and believed to be *at least* 51%.

## Summary and conclusions

Any need or drive for greater food security, in cities or on a wider scale, is highly contextual and nuanced (Clapp 2017). The presented research was conducted in respect of understanding the self-reliance, sustainability, resilience and equitability of the Leeds city food system in contrast to other modern and growing cities. The importance of such understanding of urban food security has been emphasised by recent global events. The study empirically highlighted that there is a significant gap between production of energy and nutrients with the Leeds region and the demands of its growing population, a gap that is reflected nationally. The mapping process highlighted several relationships between food system components and what appear to be spatial disparities in deprivation, food poverty and health outcomes. These observations, determined through a mapping and data comparison process that goes beyond ‘dots on a map’, requires more work to understand how the appraisals of options for expanding Leeds food system boundaries, adopting urban agriculture and/or optimising and innovating within the existing system, can help to address apparent food related inequalities. Notably, however, as was noted within several studies and clearly stated by Morgan (2009: 347) feeding cities in a fair and sustainable manner is one of the “*quintessential challenges of the twenty-first century*” that will not be met without a commitment to urban food planning.

The data gathering and mapping process demonstrated that there are many stakeholders within the Leeds food system and a multitude of activities taking place, particularly in respect of food equity and distribution to the less fortunate. As highlighted in other city region food system studies (e.g., Haenen et al. 2018), more joined up thinking is required to promote the development of a resource optimised and resilient food system that provides a healthy diet to its local populations. This includes collection and dissemination of production-consumption data. Indeed, as a significant point of note, author communications with Defra (the primary body responsible for farming in the UK) suggested that at the national level there was little knowledge of local production activities and records at the local administrative level were not formally produced or freely available. Similarly, discussion with local stakeholders from a variety of areas of system expertise highlighted the lack of knowledge of how much locally produced food is consumed in Leeds or, indeed, where it goes. This, along with a dearth of empirical knowledge of all aspects of food waste production and management, is a significant knowledge deficiency that is commonly noted within the FAO/RUAF city-region studies, and could impinge on local food security management and the likes of Morgan’s call for greater urban food planning. The adage: ‘you cannot manage what you cannot measure’, arguably applies here.

In summary, producing spatial maps of the Leeds city food system has helped to both visualise and contextualise connections between its components and the outcomes of local food production, provision and consumption. This article has largely focussed on the production of food in the area and, if necessary, whether the level and form of production taking place within the city could feed its population in a sustainable and suitably nutritious manner. This was shown, at least theoretically, not to be the case. However, going forward, the mapping process and uncovering and discussion of other traditional and innovative production opportunities, provides a roadmap for increasing the productive efficiency, equitability and resilience of the city’s food system. There is, however, much work still required on options for optimising the wider food system within the local, national and global context, particularly in regard to reducing punitive environmental impacts of the system and improving the diets and, consequently, the health of many of the less fortunate within cities across the world. It is acknowledged that growing modern cities are unlikely to ever be self-reliant in nutritious local food production. In light of climate change and other recent global events that have highlighted the fragility of urban food supply, it is however useful to be aware of just how reliant a given area is on outside provision that are, themselves, subject to the influences of a changing world. As such, it is suggested that the ontic gap - or lived reality - between how much food a city can sustainably produce and how much food is required to be self-reliant is assessed. It is recommended that this should be done alongside an exploration of options for reducing any gaps in an equitable and resource optimised circular manner, with continuous food system mapping used to engage stakeholders and monitor the impact of system shocks and interventions.

## Supplementary Information

ESM 1 (DOCX 21 kb)

## Data Availability

The source of the public data used within the study is referenced throughout. Figure data sources are provided in the supplementary material. The combination and synthesis of public datasets used within this study allows individuals to be identified. These data will be placed in the public domain when the dataset has been anonymised as necessary.

## Footnotes

1 As opposed to robust (in the systems analysis sense), which though desirable in some respects does not allow for flexible or changing diets or the ability to continue to meet dietary needs within a changing global biosphere.

2 Notably, from an industrial ecology perspective and its comparison to biological systems, there is no obvious analogue for ‘just-in-time” supply chains. Although effective from a value chain cost perspective, it reduces resource sinks and limits flows and redundancy within the system, all aspects of a biological ecosystem that contribute to its robustness and resilience (Jensen 2016).

3 Though it should be noted that anecdotal observations of fresh food losses that occurred at the height of the Covid-19 pandemic can be countered by the presence of excess frozen and long-life catering foods that becoming available to communities where suitable logistical solutions existed.

4 Yorkshire was formerly divided in 1974 into North, South, West and East Riding of Yorkshire, with Leeds sitting within the West Riding.

5 GVA - Gross Value Added – provides an indication of the gross value added by a region to products and services from the perspective of suppliers, as opposed to GDP which measures the value of final products from the demand side of production.

6 When measured on a city region basis, Greater Manchester, encompassing several cities around Manchester, has the highest GVA within the north of England (marginally higher than Leeds).

7 It should be noted that the Food Banks are complimented by numerous other subsidised or free community food schemes for children (e.g. ‘Healthy Holidays’), and older residents (e.g. ‘Over 55 s Lunch Clubs’).

8 The Land cover map layer, that depicts land parcels and what crops or other vegetation are being produced within the parcel, does not show fields smaller than 2Ha. As such the cross-reference to total cropped areas within Defra’s farm accounts was also beneficial in respect of determining omissions within the Land cover dataset.

9 The Lower Super Output Area, or LSOA, is an geographic area containing at least 1000 residents used to produce census and other small scale socioeconomic statistics.

10 In the UK, a Local Resilience Forum is a regional multiagency partnership aimed at identifying risks and planning a response to any incidents or emergencies relating to the health and safety of its communities.

11 A figure that has notably fallen from 0.45Ha in 1961 due to cultivation efficiency improvements and, notably, unsustainable intensification practices reliant on fossil fuel based agroecosystem subsidies (e.g., FAO 2011a, b).

12 Assuming 1 kg of potato (including skin) possessing 870 kcal with the greater figure derived from an increased yield of up to ~4kgm^−2^ from the stated 1.8 kg fruit and vegetable average.

13 Though not likely to demand the same volumes of fossil fuel based system subsidies (i.e. machinery diesel, fossil fuel based fertilisers, pesticides and herbicides), it should be remembered that the goal of a sustainable and resilient agroecosystem is to be self-reliant and not to be dependent on the same material and energy subsidies that drives down the efficiency of industrial farming.

14 Indeed, it is noted that the city of Detroit, in the United States, is an exemplar in the communal use of what could be described as derelict or abandoned land following the city’s historic and recent economic declines (e.g. Pfleger 2018).

15 It is noted that most commonly used fish in aquaponics, tilapia, does not possess a good fat profile compared to the likes of wild salmon or other oily fish that are recommended within a healthy diet, i.e. mackerel and herring.

16 The ability for innovative multi-spatial urban farming systems to produce crop yields at a scale meaningful to the demands of a city continues to be debated, with calls for more research in this area being recently made (i.e., Beacham et al. 2019).
