# Supplementary material for: Mapping the production-consumption gap of an urban food system: an empirical case study of food security and resilience
Source: Food Secur. 2021 Feb 8;13(3):551–70. doi: 10.1007/s12571-021-01142-2 (PMC7868171; doi:10.1007/s12571-021-01142-2)
Supplement: Supplementary file 1 — (DOCX 21 kb) [file 12571_2021_1142_MOESM1_ESM.docx]

**MAPPING THE PRODUCTION-CONSUMPTION GAP OF AN URBAN FOOD SYSTEM:**

**AN EMPIRICAL CASE STUDY OF FOOD SECURITY AND RESILIENCE**

| **Map Layer** | **Source/Reference** | **Map Layer Description and Editing Notes** |
| --- | --- | --- |
| ***Polygon Map Layers*** | | |
| Leeds City Region | Contains Ordnance Survey data © Crown copyright and database right 2020 | Boundary layer of towns and cities which combine to form the Leeds City Region.  Created from OS Boundary Line layer. |
| LCR Green Space | As Above | Map of green spaces in LCR that can be mapped by function, i.e. tennis court, playing field etc. |
| Leeds Green Space | As Above | As above, cropped to Leeds city boundary. |
| LCR Wards | As Above | Created from OS Boundary Line data. |
| Leeds | As Above | Boundary map of Leeds. |
| Hydrogeology | Contains British Geological Survey materials © NERC 2020 | Data layer provides information on productivity of locally important aquifers.  Mapped data layer clipped to LCR boundary. |
| Flood Risk | Geological Map Data BGS © UKRI 2020 | Map layer provides an indication of the risk (high/low) of, in this case, flooding from fluvial water sources and their respective zoning classifications.  Data layer clipped to LCR boundary. |
| Crops 2016 | © NERC (CEH) | Layer provides an indication of land use type for parcels of land of least **2ha** in scale. Crop types and land configuration are determined by annual farmer declarations to RPA, aerial photography and LIDAR. For data accuracy see: <https://www.ceh.ac.uk/ceh-land-cover-plus-crop-map-quality-assurance> |
| Crops 2017 | © NERC (CEH) | As Above. |
| Crops 2018 | © NERC (CEH) | As Above. |
| Allotments | Contains Ordnance Survey data © Crown copyright and database right 2020 | A map layer of all allotments cropped to those within the Leeds city boundary.  Total Ha of each allotment polygon calculated using ArcGIS ‘calculate geometry’ function. |
| Leeds Soils | Contains British Geological Survey materials © NERC 2020 | Data layer provides information on the quality and distribution of the variety of soil types found within Leeds. |
| Warehouses/Storage | Contains UKBuildings data © Geomni 2020 | Data layer providing information on the location, age and total working area of all warehouses and storage buildings within Leeds (for use in spatial analysis of potential food hubs and identification of innovative production opportunities). |
| Derelict Buildings | Contains UKBuildings data © Geomni 2020 | Data layer providing information on the location, age and total working area of all currently derelict or disused buildings within Leeds (for use in spatial analysis of potential food hubs and identification of innovative production opportunities). |
| ***Polygon Attribute Data*** | | |
| Ward Name | Contains Ordnance Survey data © Crown copyright and database right 2020 | Created from cropped from OS Boundary Line map layer. |
| Hectares | - | Created using ArcGIS geometry function. |
| Population | Contains public sector information licensed under the Open Government Licence v3.0.  <https://www.gov.uk/government/statistics/english-indices-of-deprivation-2019> and <https://observatory.leeds.gov.uk/deprivation/>  September 26th 2019 | Extracted from MID data.  Data provides total population and age brackets: 0-15, 16-64, 65> |
| Multiple Index of Deprivation | See Above | The MID is a weighted indicator of relative deprivation comprised of measures of: Income 22.5%, Employment 22.5%, Education and Skills 13.5%, Health and Disability 13.5%, Crime 9.3%, Barriers to Housing 9.3%, Living Environment 9.3%.  Attribute table includes government LSOA scores plus relative Leeds ward rankings based on summation of each wards’ deprivation score. Table also includes calculation of average ward deprivation decile, i.e. 1 - 10. |
| Child Obesity | National Child Measurement Programme dataset, NHS Digital  <https://digital.nhs.uk/services/national-child-measurement-programme/>  2018 | Map layer shows percentage of children classed as being overweight and obese at school reception and at year 6. Data for 2013 and 2018 were extracted and mapped. |
| 5aDay | Health Leeds: A Food Strategy for Leeds 2006-2010  <https://leedsfoodpartnership.files.wordpress.com/2016/07/leeds-food-strategy-final-leeds-food-matters.pdf> (DH/ONS quoted as source).  2010 | For each council ward the percent of children and adults consuming 5 portions of fruit and vegetables are provided (N.B. reference figures provided are for 2003 council wards – data was allocated to current wards based on average of current overlapping wards as such, caution should be taken with this indicator estimates were in general 1-2% different to original data and ward allocations). |
| Diabetes | Public health data recorded by Leeds GP practices: Open Government Licence v3.0. <https://data.gov.uk/dataset/95976d59-2d18-4c5a-91c3-98b2be5d1617/public-health-data-gp-recorded-conditions-leeds> | Directly Age Standardised Rates (DASR) per 100,000. Age standardised rates compensate for differing age structures by weighting them to meet the European Standard Population (2013). Rates can then be compared for different areas, or even across area types.  N.B. Headingley 2013-2017 data assigned to Headingley and Hyde Park 2018 data/ward (likewise for: City & Hunslet > Hunslet & Riverside; Hyde Park & Woodhouse > Little London & Woodhouse).  Mapped dataset is for DSR 2013-2018. 95% CIs are not shown within mapped datasets. |
| Heart Disease | As Above | As Above |
| Obesity | As Above | As Above |
| Building Count | - | Polygons for OS Buildings layer were converted to (internal) point features for each discrete building and summed for each ward. |
| Building Area (m2) | - | For each OS Building polygon its area in m2 was calculated and assigned to its ward and totalled via geometry function. |
| Public Houses and Bars | - | Total number of pubs and bars from FoodPremisesFSA layer within each ward. |
| Restaurants and Cafes | - | Total number of restaurants from FoodPremisesFSA layer within each ward. |
| Supermarkets | - | Total number of supermarkets from FoodPremisesFSA layer within each ward. |
| Takeaways | - | Total number of takeaways from FoodPremisesFSA layer within each ward. |
| ***Line/Point Layers*** | | |
| Food Premises | Contains public sector information licensed under the Open Government Licence v3.0.  <https://ratings.food.gov.uk/open-data/en-GB>  February 2020 | Dataset showing food hygiene ratings and location of food serving/preparation businesses. Businesses include restaurants, pubs, cafés, takeaways, hotels and other places consumers eat, supermarkets and other food shops.  Data georeferenced via: Lat/Long Batch Convertor. |
| Supermarkets | See Above | Point data layer showing location of all super/hypermarkets within and extracted from FoodPremisesFSA layer. |
| Restaurants/Cafes | See Above | Point data layer showing location of all restaurants and cafes within and extracted from FoodPremisesFSA layer. |
| Pubs/Bars | See Above | Point data layer showing location of all pubs, bars and clubs within and extracted from FoodPremisesFSA layer. |
| Takeaways | See Above | Point data layer showing location of all takeaways and sandwich shops within and extracted from FoodPremisesFSA layer. |
| Distributors | See Above | Point data showing location of all FSA registered food distributors/storage sites. |
| Manufacturers | See Above | Point data showing location of food processors and packers as extracted from the FoodPremisesFSA layer. |
| Food Banks | Food bank information from Sonja Woodcock, Leeds and: <https://leedsnorthandwest.foodbank.org.uk/> and <https://leedssouthandeast.foodbank.org.uk/> | Point Data showing the location of each food bank within the Leeds city metropolitan area. The attribute table for the layer provides information on all banks donation drop off points and the days that the banks are accessible to the public. |
| Food Bank Drop Off | As Above | Point data showing the location of each food bank donation drop off point. |
| Food Bank Routes | As Above | Line data showing the distribution network of food bank and donation drop off points. |
| Pollution | For LCC and Headingley: © Crown 2020 copyright Defra via uk-air.defra.gov.uk, licenced under the Open Government Licence (OGL)  <https://uk-air.defra.gov.uk/data/>  Data for all others sites: [data.gov.uk/dataset/ratified-air-quality-nitrogen-dioxide](https://data.gov.uk/dataset/e3c001d1-f3d2-44db-bc30-833cb38d553c/ratified-air-quality-nitrogen-dioxide) and [cleanairleeds.co.uk/2018.pdf](https://cleanairleeds.co.uk/sites/default/files/Leeds%20ASR%202018.pdf) |  |
| Rethink Food | Information on schools engaged in RethinkFood programme received from Sonja Woodcock: <https://www.rethinkfood.co.uk/education/> | Point data layer for schools in Leeds receiving weekly surplus food from RethinkFood for selling via market stalls to local families. RF objectives are: 1. Remove hunger as a barrier to learning; 2. Reduce the amount of perfectly edible food that is wasted; 3. Educate children and families to make better food choices that result in improved health and wellbeing AND a sustainable future. |
| Roads | Contains Ordnance Survey data © Crown copyright and database right 2020 | Line data for the wider Leeds city region road network, with separate layer only showing primary roads within the city. |
